# Supplementary material for: PS-NPs Induced Neurotoxic Effects in SHSY-5Y Cells via Autophagy Activation and Mitochondrial Dysfunction
Source: Brain Sci. 2022 Jul 20;12(7):952. doi: 10.3390/brainsci12070952 (PMC9321807; doi:10.3390/brainsci12070952)
Supplement: Supplementary file 1 [file brainsci-12-00952-s001.zip › brainsci-1787761-SI.pdf]

PS-NPs induced neurotoxic effects in SHSY-5Y cells via autophagy activation and mitochondrial dysfunction

Qisheng Tang [†], Tianwen Li [†], Kezhu Chen, Xiangyang Deng, Quan Zhang, Hailiang Tang, Zhifeng Shi, Tongming Zhu *, Jianhong Zhu *

Fudan University Huashan Hospital, Department of Neurosurgery, National Center for Neurological Disorders, National Key Laboratory for Medical Neurobiology, Shanghai Key Laboratory of Brain Function and Regeneration, Institutes of Brain Science, MOE Frontiers Center for Brain Science, Shanghai Medical College-Fudan University, 12 Wulumuqi Zhong Rd., Shanghai 200040, China.

* **Corresponding Author:** jzhu@fudan.edu.cn ztm1911@126.com

[†] Both authors contributed equally to this work.

Materials and Method

Polystyrene nanoparticles (PS-NPs) material

Commercially PS-NPs beads were purchased from Janus New-Materials (Nanjing, China). The detailed parameters are as follows: diameter, 50 nm; solid content, 5%, w/v; coefficient of variation (c.v), ≤5%.

MTT assay

SHSY-5Y cells were seeded in a 96-well culture plates at 37 °C, under a humidified atmosphere in a CO_2 incubator. The SHSY-5Y cells were treated with different concentrations of PS-NPs (20, 50, 100, 200, and 500 mg/L) for 24 h. Follwing PS-NPs treatment, 100 mL of MTT reagent (5 mg/mL) was added and co-incubated with culture medium for another 4 h. Then, the supernatant was discarded, and 150 ml of DMSO was added to culture plates, which were gently shaken for 3 min in dark on a shaker platform to dissolve the formazan crystals. The absorbance of formazan were recorded at 570 nm using a microplate reader (Spark® 20M Multimode Microplate Reader, Tecan, Switzerland).

LDH release assay

SHSY-5Y cells seeded in a 96-well culture plates were treated with different concentrations of PS-NPs (20, 50, 100, 200, and 500 mg/L) for 24 h. LDH reagent (1:100) was added to culture medium and co-incubated at 37 °C for another 1 h. Then, 120 µL of RPMI 1640 medium supernatant was transferred to a new 96-well plate, and 60 µL of LDH detection reagent were added. After further incubation for 30 min in dark, absorbance at 490 nm was recorded with a microplate reader (Spark® 20M Multimode Microplate Reader, Tecan, Switzerland).

ROS measure

SHSY-5Y cells seeded in a 6-well culture plates were treated with PS-NPs (20, 50, 100, 200, and 500 mg/L) for 24 h. SHSY-5Y cells were then incubated with the fluorescent probe DCFH-DA (10 µM) for 30 min in dark. After that, SHSY-5Y cells were rinsed three times using PBS buffer. Cell imager (ZOE™ Fluorescent Cell Imager, BIO-RAD, USA) was used to record fluorescence images with excitation at 480 nm and emission at 525 nm. Image-Pro Plus (Medical Cybernetics, Version 6.0) was used to quantify the fluorescent intensity. The oxidative stress inducer tBHP (100 µM) was used as a positive control for oxidative stress detection.

MMP (Δψm) measure

SHSY-5Y cells seeded in a 6-well culture plates were treated with PS-NPs (20, 50, 100, 200, and 500 mg/L) for 24 h. SHSY-5Y cells were then incubated with a fluorescent probe Rh 123 (10 μM) for 30 min in dark. After washing three times using PBS buffer, cellular fluorescence images was recorded with excitation at 507 nm and emission at 529 nm by Cell imager (ZOE™ Fluorescent Cell Imager, BIO-RAD, USA). Image-Pro Plus (Medical Cybernetics, Version 6.0) was used to quantify the fluorescent intensity.

Intracellular calcium ion (Ca^{2+}) content measure

SHSY-5Y cells seeded in 6-well culture plates were treated with PS-NPs (20, 50, 100, 200, and 500 mg/L) for 24 h. SHSY-5Y cells were then incubated with a fluorescent probe Fluo-3AM (5 μM) for 1 h in dark. After that, SHSY-5Y cells were washed three times using PBS buffer. Cell imager (ZOE™ Fluorescent Cell Imager, BIO-RAD, USA) was used to record the fluorescence images with excitation at 506 nm and emission at 526 nm. Image-Pro Plus (Medical Cybernetics, Version 6.0) was used to quantify the fluorescent intensity.

ATP level measure

After treatment with PS-NPs (20, 50, 100, 200, and 500 mg/L) for 24 h, culture medium was removed and lysis solution (1:10) was added. After lysis, the supernatant was collected by centrifuging at 12,000 g for 5 min at 4 °C. ATP detection working solution was added and incubated with supernatant for 3-5 min. The relative light unit (RLU) was measured by a microplate reader (Spark® 20M Multimode Microplate Reader, Tecan, Switzerland).

Apoptosis assay

After exposure to PS-NPs (20, 50, 100, 200, and 500 mg/L) for 24 h, the culture medium was removed and EDTA-free trypsin was added. After washing two times with PBS, cell pellets were collected with centrifuge at 3,000 g for 5 min. Then, 100 ml 1 × binding buffer was applied to resuspend SHSY-5Y cells. The cell suspensions were incubated with 5 ml Annexin V-FITC and 5 mL PI staining solution in the dark for 10 min at room temperature. Finally, 400 ml 1 × binding buffer were added, and Flow Cytometer (CytoFLEX s, Beckman Coulter, USA) was used to analyze the apoptosis.

Reverse transcription quantitative polymerase chain reaction (RT-qPCR)

After treatments of 100 and 200 mg/L PS-NPs for 12 h, SHSY-5Y cells were lysed using TRIZOL reagent according to the instructions to prepare the total RNA. The first strand of cDNA was reverse-transcribed from the total RNA with a reverse transcription kit (TOYOBO, Osaka, Japan). The cDNA was used as a template for real-time quantitative PCR amplification with SYBR Green PCR Master Mix (Toyobo, Osaka, Japan). The amplification was started by 10 min incubation at 95 °C to denature the template cDNA and activate Taq polymerase, then followed by 40 cycles of denaturation at 95 °C for 15 s, annealing and extension at 63 °C for 30 s. Each qPCR measurement was run with triplicate repeats for each cDNA sample. No-template controls (NTC) were included in each measurement. The ΔΔCt method was used to quantify the gene expression levels after normalization to the internal reference gene (GAPDH). The primers of Beclin-1 were purchased from Sunny Biotechnology (Shanghai, China). (Forward Primer: CTGGTAGAAGATAAAACCCGGTG, Reverse Primer: AGGTAGAGCGTGGACTATCCG.)

Western Blotting

After treatments of 100 and 200 mg/L PS-NPs for 24 h, the total protein was collected and subjected to sodium dodecyl sulfate-polyacrylamide gel electrophoresis. The protein samples were transferred to the polyvinylidene fluoride membrane (Sigma, MO, USA). The primary and corresponding secondary antibodies were then incubated with protein samples according to the manufacturer's instructions. Finally, blots were visualized using chemiluminescence, and the optical density of each band was quantified using the ChemiDoc imaging system (Bio-Rad, CA, USA).

Results

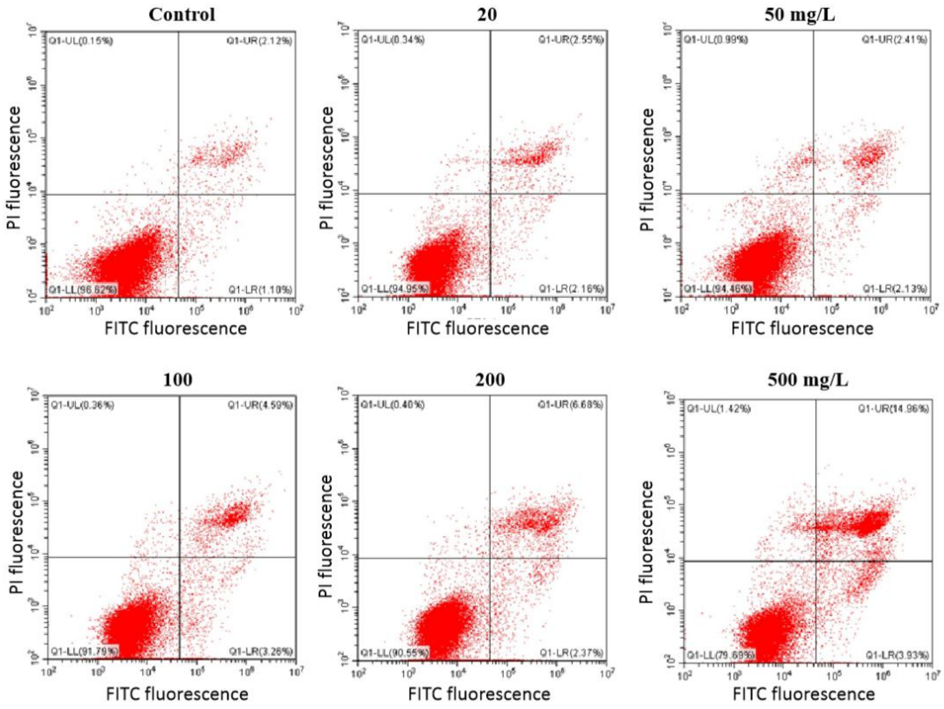

Figure S1. The flow cytometry dot plot for Figure 2A

The vertical axis refers to the propidium iodide channel, and the horizontal axis is the annexin V-FITC channel.

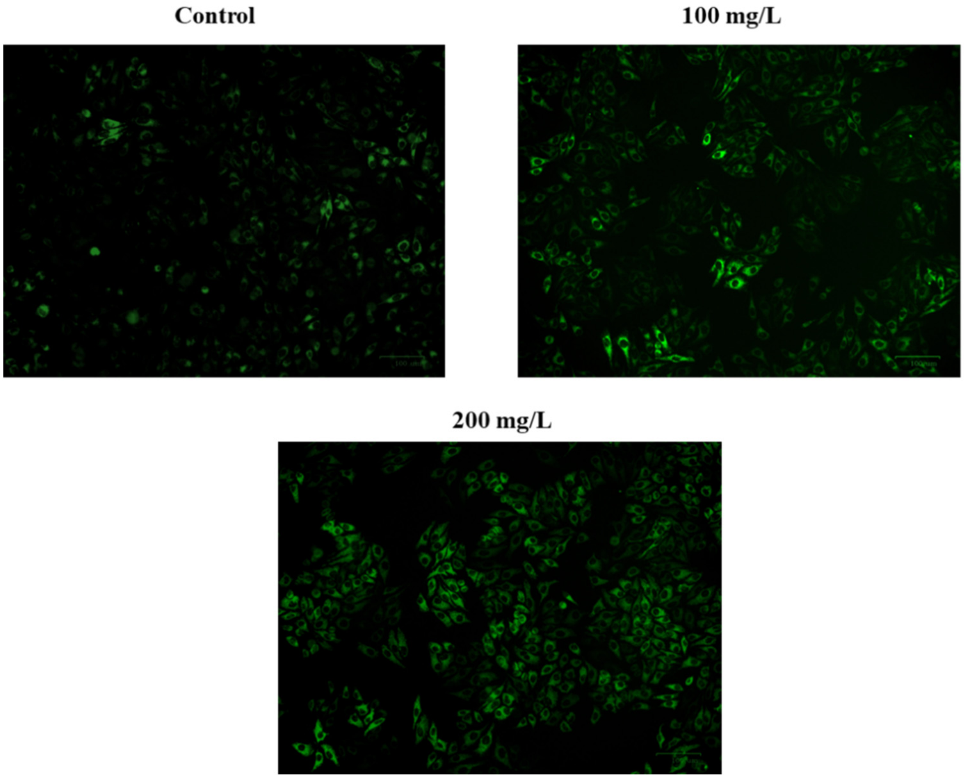

Figure S2. The large-size images of Figure 2B

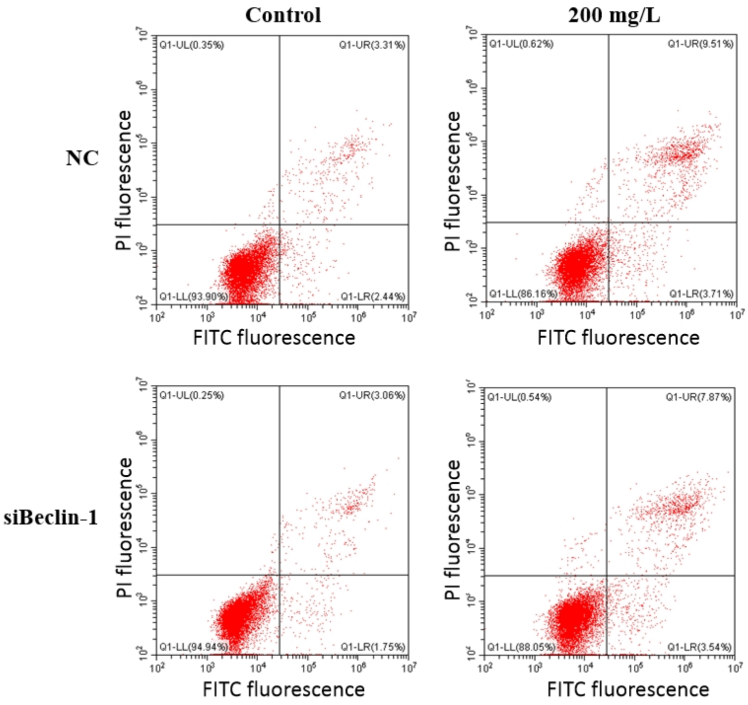

Figure S3. The flow cytometry dot plot for Figue 4C

The vertical axis refers to the propidium iodide channel, and the horizontal axis is the annexin V-FITC channel.

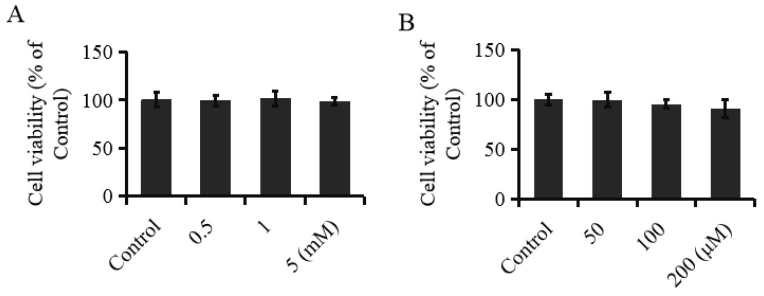

Figure S4. The effect of NAC and tBHP on cell viability

(A): SHSY-5Y cells were treated with NAC (0.5, 1, 5 mM) for 4 h, cell viability was measured with MTT assay. (B): SHSY-5Y cells were treated with tBHP (10, 100, 200 μM) for 1 h, cell viability was measured with MTT assay.

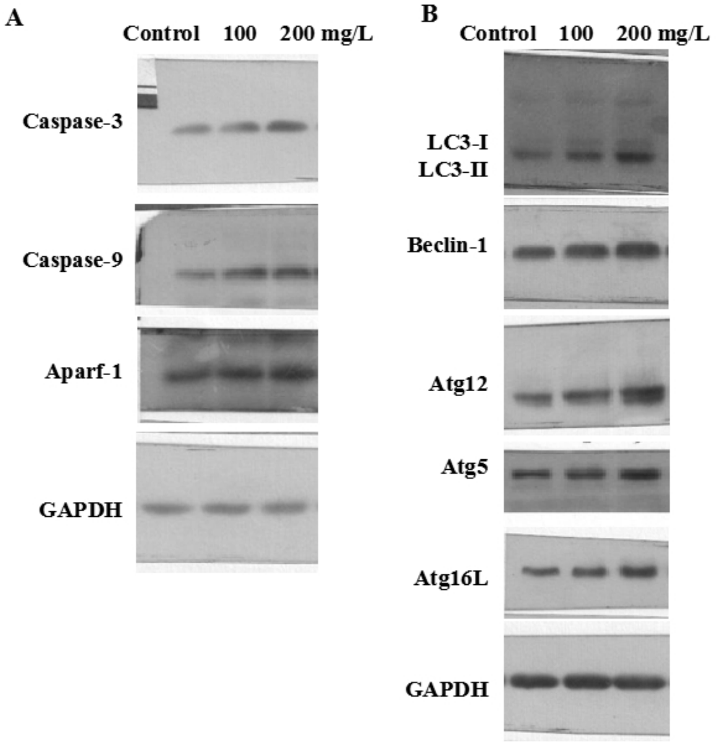

Figure S5. (A): The full images of Figure 2C. (B): The full images of Figure 3A.

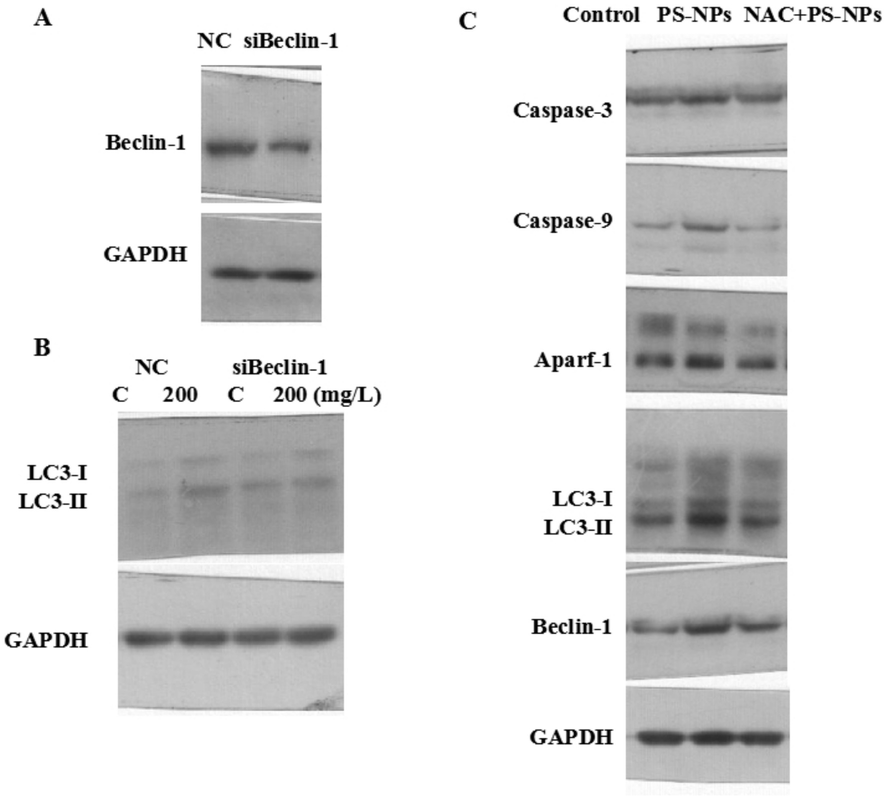

Figure S6. (A): The full images of Figure 4A. (B): The full images of Figure 4B. (C): The full images of Figure 5B.
